# Conference Calendar

**DOI:** 10.1002/cfg.171

**Published:** 2002-06


					Conference Calendar
September-December 2002
Animal models
16th Intl. Mouse Genome Conference -
November 17-20
http://imgs.org/cal.html
CHI - Application of Genomics to Animal
Models for Pharmaceutical Studies -
November 20-21
http://www.healthtech.com/2002/anm/index.htm
Bacteria
IP - Integrative Approaches in Microbial
Pathogenesis - November 13-16
http://www.pasteur.fr/infosci/conf/CLP4.html
Bioinformatics
2002 CSHL/Wellcome Trust - Genome
Informatics - September 4-8
http://meetings.cshl.org/2002infouk.htm
CHI - Microarray Data Analysis - September
10-11
http://www.healthtech.com/2002/mda/index.htm
CCP11 - Genes, Proteins and Computers VII
- September 11-13
http://www.hgmp.mrc.ac.uk/CCP11/CCP11conference_
GPCVII.jsp
CHI - Protein Informatics - September 12-13
http://www.healthtech.com/2002/pnf/index.htm
CHI - Data Visualization and Interpretation -
September 12-13
http://www.healthtech.com/2002/dvs/index.htm
European Conference on Computational
Biology 2002 - October 6-9
http://www.eccb2002.de/
ESF - Ontology for Biology - November 7-8
http://www.functionalgenomics.org.uk/sections/activitites/
2002/Bernardi/Bernardi.htm
13th Intl. Conference on Genome Informatics
(GIW 2002) - December 16-18
http://giw.ims.u-tokyo.ac.jp/giw2002/
3rd WSEAS Intl. Mathematics And Computers
In Biology And Chemistry - December 19-21
http://www.wseas.org/conferences/2002/tenerife/mcbc/
Comparative genomics/evolutionary
genomics
CSHL - Comparative Plant Genomics -
December 12-15
http://meetings.cshl.org/2002Arab.htm
Functional genomics
EMBL/EMBO Symposium - Functional
Genomics: The Future of Biology - October
13-16
http://www.embl-heidelberg.de/conferences/
omics2002/
CHI - Functional Genomics - November
18-19
http://www.healthtech.com/2002/fgn/index.htm
Comparative and Functional Genomics
Comp Funct Genom 2002; 3: 289-291.
Published online 2 May 2002 in Wiley InterScience (www.interscience.wiley.com). DOI: 10.1002/cfg.171
Copyright # 2002 John Wiley & Sons, Ltd.
General genomics
World Genomics Symposium - September
18-20
http://www.world-genomics.com/V26/index.cvn?id=
10000&p_navID=1
10th
Annual Atlas Venture Life Sciences
Conference - September 29-October 1
http://www.atlasventure.com/lsc/
TIGR - 14th Intl. Genome Sequencing and
Analysis - October 2-5
http://www.tigr.org/conf/gsac/main.htm
GenomeX 2002 - October 14-16
http://www.pharma-rd.net/genomex2002/
Human
1st Intl. Human Genome Variation Society
Annual General Meeting - October 15
http://www.genomic.unimelb.edu.au/mdi/meetings/
balt.html
ASHG - 52nd
Annual Meeting - October
15-19
http://www.faseb.org/genetics/ashg/2002meeting/
index.html
4th HUGO Pacific Meeting + 5th Asia-Pacific
Conf. On Human Genetics - October 27-30
http://www.mu-st.net/hugothai/
CSHL - Human Origins & Disease - October
30-November 3
http://meetings.cshl.org/2002evol.htm
Metabolomics
CHI - Metabolic Profiling - December 2-3
http://www.healthtech.com/2002/mbp/index.htm
CHI - Metabolic Engineering - December 2-3
http://www.healthtech.com/2002/meg/index.htm
Pharmacogenomics
IBC - Model Organisms for Drug Discovery -
August 8-9
http://www.lifesciencesinfo.com/2713
ESF - The future of human biobanks -
September 12-13
http://www.functionalgenomics.org.uk/sections/activitites/
2002/Alarc-n-Riquelme/info.htm
IP Euroconferences - Pharmacogenomique
(Pharmacogenomics) 2 - October 17-18
http://www.pasteur.fr/applications/euroconf/
pharmacogen/index.html
CHI - Chemical Genomics/Chemogenomics -
November 18-19
http://www.healthtech.com/2002/chg/index.htm
CHI - High-Throughput Target Validation -
November 20-21
http://www.healthtech.com/2002/tvd/index.htm
Plants
Intl. Rice Congress - September 16-20
http://www.irri.org/IRC2002/introduction.htm
3rd
Annual GARNet Functional Genomics
Meeting - September 17-18
http://www.york.ac.uk/res/garnet/usermeet.htm
1st
Plant Genomics European Meeting
(Plant-GEM) - September 29-October 2
http://www.plant-gems.org/Eins.html
Intl. Symp. Plant Species-level Systematics -
November 13-15
http://www.nationaalherbarium.nl/symposium2002/
Proteomics
IIR - BioArrays Europe - September 27-29
http://www.bioarrayseurope.com
290 Conference Calendar
Copyright # 2002 John Wiley & Sons, Ltd. Comp Funct Genom 2002; 3: 289-291.
IIR - Genomics and Proteomics Congress -
October 28-30
http://www.iir-genomics.com
Structural genomics
IAPSAP - 14th
Methods of Protein Structure
Analysis (MPSA2002) - September 8-12
http://www.mpsa2002.ibv.csic.es/
Systems biology
3rd Intl. Conference on Systems Biology
(ICSB2002) - December 13-15
http://www.ki.se/icsb2002/
Transcriptomics
CSHL - Translational Control - September
10-15
http://meetings.cshl.org/2002transc.htm
SRI - Microarray Technologies Summit -
September 23-24
http://www.srinstitute.com/part_iter_site_page.cfm?
iteration_id=387
IBC - BioDigital 2002 - October 9-11
http://www.biodigital.de/
IBC - Chips to Hits - October 27-31
http://www.chipstohits.com/
Biotech industry
5th
European Biotechnology Symposium -
September 29-October 1
http://www.bioconferences.com/ebs/
IIR - International Biotech - November 19-20
http://www.internationalbiotech.com/
Key
ASHG - American Society for Human Genetics
[http://www.ashg.org/]
CCP11 - Collaborative Computing Project 11
[http://www.hgmp.mrc.ac.uk/CCP11/]
CHI - Cambridge Healthtech Institute [http://
www.healthtech.com/]
CSHL - Cold Spring Harbor Laboratory [http://
www.cshl.org/]
ESF - European Science Foundation [http://www.
esf.org]
IAPSAP - International Association for Protein
Structure Analysis and Proteomics [http://www.med.
virginia.edu/medicine/basic-sci/micr/fox/iapsap/iapsap.
html]
IBC - IBC Conferences [http://www.ibcusa.com]
IIR - IIR Conferences [http://www.iir-conferences.
com/]
IP - Institut Pasteur [http://www.pasteur.fr]
SRI - Strategic Research Institute [http://www.
srinstitute.com]
TIGR - The Institute for Genome Research [http://
www.tigr.org]
WSEAS - World Scientific and Engineering Acad-
emy and Society [http://www.wseas.org/]
The Conference Calendar is a list of forthcoming conferences of interest to our readership. We provide the
title, dates and web address of each conference, to enable readers to find out more information. Inclusion
does nor imply endorsement by the Journal. If you know of a relevant conference, please contact the
Managing Editor.
Conference Calendar 291
Copyright # 2002 John Wiley & Sons, Ltd. Comp Funct Genom 2002; 3: 289-291.

				

